# Long Non-Coding RNAs and Circular RNAs in the Pathobiology of T-Cell Lymphoma

**DOI:** 10.3390/cancers18162535

**Published:** 2026-08-07

**Authors:** Shahed Azzam Ahmed Abdullah, Richard Flavin

**Affiliations:** 1 Department of Histopathology, Trinity College Dublin, D02 PN40 Dublin, Ireland; flavinr@tcd.ie; 2 Division of Hematology and Oncology, Department of Internal Medicine, UT Southwestern Medical Center, Dallas, TX 75390, USA; 3 Department of Histopathology, St. James’s Hospital, D08 NHY1 Dublin, Ireland

**Keywords:** PTCL, T-cell lymphoma, NKTL, TLBL, CTCL, ALCL, linear non-coding RNAs, circular non-coding RNAs, long non-coding RNAs, gene expression

## Abstract

Peripheral T-cell lymphomas (PTCLs) are a heterogeneous group of clinically aggressive mature T-cell and natural killer (NK)-cell neoplasms that account for approximately 10–15% of all non-Hodgkin lymphomas in Western countries . The most common subtypes include extranodal NK/T-cell lymphoma (ENKTL), nodal T-follicular helper cell lymphomas, peripheral T-cell lymphoma, not otherwise specified (PTCL-NOS), anaplastic large cell lymphoma (ALK-positive and ALK-negative), and T-cell lymphoblastic lymphoma. Non-coding RNAs (ncRNAs) constitute the majority of the human transcriptome and play critical roles in regulating gene expression, cellular proliferation, differentiation, migration, and apoptosis. Among these, long non-coding RNAs (lncRNAs) and circular RNAs (circRNAs) have emerged as key regulators of lymphomagenesis and disease progression in PTCLs. These molecules modulate diverse oncogenic pathways through chromatin remodeling, transcriptional regulation, competing endogenous RNA activity, and interactions with RNA-binding proteins, thereby influencing proliferation, immune evasion, treatment resistance, and clinical outcomes. Representative examples include the lncRNA TCLlnc1, which promotes PTCL progression through activation of transforming growth factor-β (TGF-β) signaling, and the circRNAs circKIF4A, circADARB1, and circ-LAMP1, which regulate miRNA-dependent signaling networks involving PDK1/BCL11A, STAT3, and DDR2, respectively. In this review, we summarize the current understanding of the biological and clinical roles of lncRNAs and circRNAs in PTCL and related T-cell and NK-cell neoplasms and highlight their potential as diagnostic and prognostic biomarkers as well as therapeutic targets. We also discuss recent advances and future directions for integrating ncRNA-based approaches into precision medicine for T-cell lymphoma.

## Simple Summary

Peripheral T-cell lymphomas (PTCLs) are rare but aggressive blood cancers that often have poor outcomes because effective biomarkers and targeted therapies remain limited. Recent research has identified long non-coding RNAs (lncRNAs) and circular RNAs (circRNAs) as important regulators of gene expression that contribute to lymphoma development, progression, and treatment resistance. These RNA molecules also show promise as minimally invasive biomarkers for diagnosis, prognosis, and disease monitoring, while representing potential therapeutic targets. This review summarizes the current understanding of the biological functions of lncRNAs and circRNAs in T-cell and natural killer (NK)-cell lymphomas, highlights their clinical relevance, and discusses future opportunities for integrating RNA-based biomarkers and therapies into precision medicine.

## 1. Introduction

Peripheral T-cell lymphomas (PTCLs) are a heterogeneous group of rare and clinically aggressive mature (post-thymic) T-cell and natural killer (NK)-cell neoplasms that account for approximately 10–15% of all non-Hodgkin lymphomas in Western countries and occur more frequently in parts of Asia and Latin America [1,2,3]. Contemporary epidemiologic studies continue to demonstrate marked geographic variation in the incidence and molecular characteristics of T-cell and NK-cell lymphomas, particularly the higher prevalence of NK/T-cell neoplasms in Asia [4]. PTCLs generally affect older adults, with a median age at diagnosis of approximately 60 years and a slight male predominance [3]. Frequently encountered entities include peripheral T-cell lymphoma, not otherwise specified (PTCL-NOS), nodal T-follicular helper (TFH) cell lymphomas, extranodal NK/T-cell lymphoma (ENKTL), anaplastic large cell lymphoma (ALCL), cutaneous T-cell lymphomas (CTCLs), and T-cell lymphoblastic lymphoma (T-LBL) [1,5,6]. These entities together account for the majority of mature T-cell and NK-cell neoplasms encountered in clinical practice. Despite substantial advances in molecular characterization and targeted therapy, effective biomarkers for diagnosis, risk stratification, and treatment selection remain limited.

The current classification of lymphoid neoplasms is based on two complementary systems: the 2022 International Consensus Classification (ICC) along with the fifth edition of the WHO Classification of Haematolymphoid Tumours (WHO-HAEM5) [6,7] (Table 1). Both frameworks emphasize the marked biological and clinical heterogeneity of PTCLs and underscore the importance of integrating morphological, immunophenotypic, genetic, and molecular features into routine diagnosis. Despite considerable molecular research, there remains a critical need for the development of effective predictive biomarkers and therapeutic targets for aggressive forms of T-cell lymphoma.

Epigenetic dysregulation is a hallmark of many PTCL subtypes [7]. Beyond recurrent alterations involving DNA methylation, histone modification, and chromatin remodeling pathways, accumulating evidence suggests that non-coding RNAs (ncRNAs) contribute substantially to lymphoma development and progression. Emerging evidence indicates that ncRNAs shape anti-tumor immunity by regulating immune checkpoint expression, antigen presentation, and interactions within the tumor microenvironment [8]. Protein-coding messenger RNAs comprise only a small fraction of the human transcriptome, whereas the majority consists of ncRNAs with diverse regulatory functions [9].

**Figure 1:**
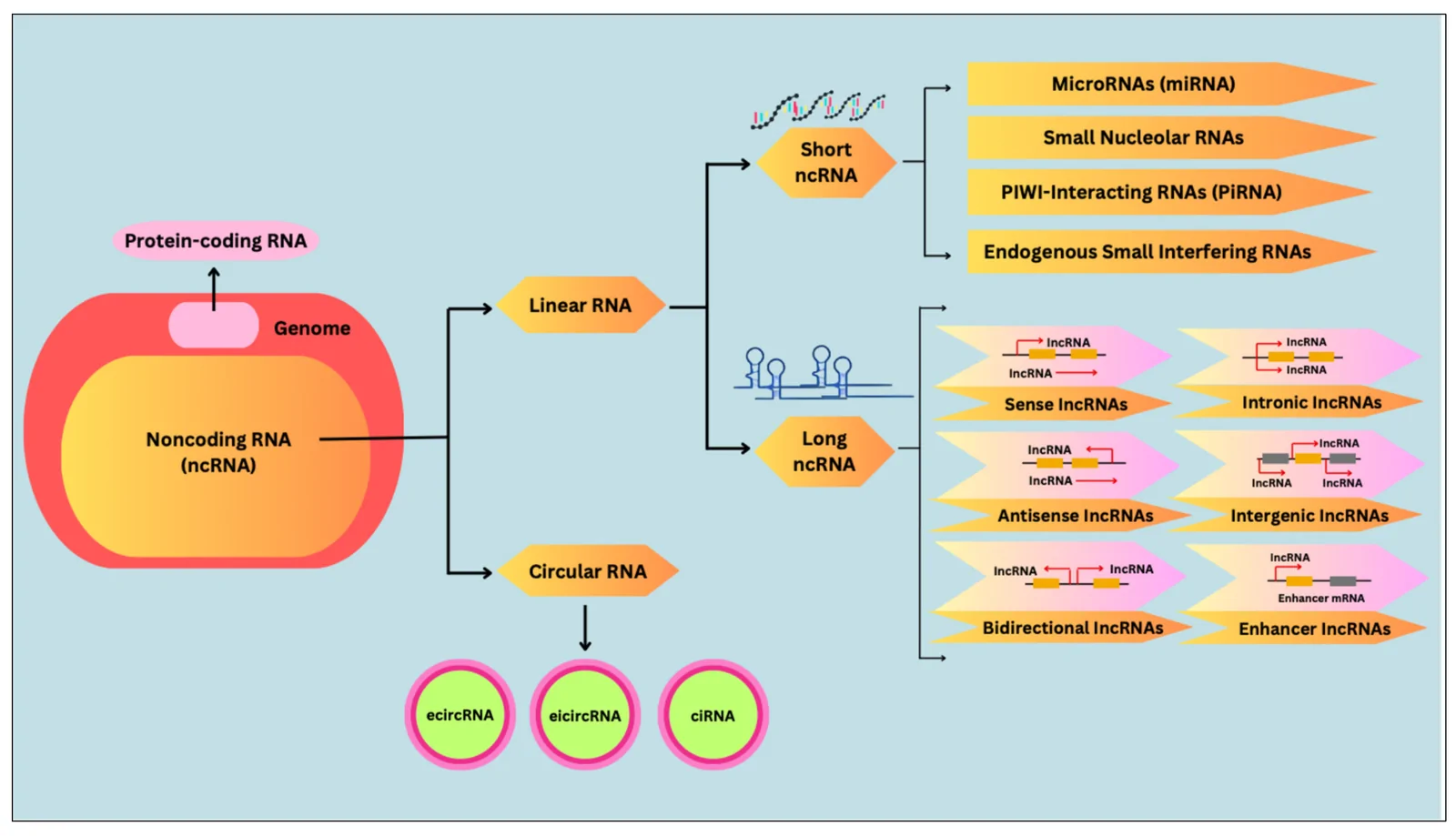
Classification of non-coding RNAs (ncRNAs) in the human transcriptome.

NcRNAs can be broadly classified according to molecular structure into linear RNAs and circular RNAs (circRNAs) [10]. CircRNAs are covalently closed RNA molecules lacking 5′ caps and 3′ poly(A) tails. CircRNAs regulate gene expression through multiple mechanisms, including microRNA (miRNA) sponging, competing endogenous RNA (ceRNA) activity, interactions with RNA-binding proteins, modulation of transcription, and, in selected cases, translation of short functional peptides [11]. Linear ncRNAs can be divided into short ncRNAs and long non-coding RNAs (lncRNAs), which are conventionally defined as transcripts longer than 200 nucleotides with limited or no protein-coding potential [10,12]. Small non-coding RNAs also regulate immune-cell differentiation, cytokine signaling, and immune evasion, further supporting the broader contribution of RNA-based mechanisms to tumor immunity [13]. Examples of short ncRNAs include microRNAs (miRNAs), small nuclear RNAs (snRNAs), small nucleolar RNAs (snoRNAs), PIWI-interacting RNAs (piRNAs), and endogenous small interfering RNAs (siRNAs) [7,11,14,15] (Figure 1).

LncRNAs are non-coding RNAs, approximately more than 200 nt in length, and are involved as main mediators of gene expression [16]. Like mRNAs, lncRNAs are transcribed by polymerase II, become spliced, 5′ capped and then undergo polyadenylation. Unlike mRNAs, lncRNAs are not translated into protein [16]. LncRNAs are transcribed from intergenic, intronic, antisense, enhancer-associated, and bidirectional genomic regions [11,14] (Figure 1). Classification of lncRNAs is according to their orientation and position in the genome including sense, antisense, intergenic, intronic and bidirectional [10] (Figure 1). Unlike sense lncRNAs, which originate from the same strand as protein-coding genes, antisense lncRNAs are transcribed from the opposite strand and frequently overlap with exonic regions of protein-coding loci [16]. Intronic lncRNAs arise from intronic regions and do not overlap with exonic sequences [16]. Some lncRNAs sequences are located within protein-coding genes including bidirectional, transcribed from the opposite DNA stand, and intergenic lncRNAs, transcribed intergenically from both strands [16]. These lncRNAs influence gene expression through mechanisms involving chromatin remodeling, transcriptional regulation, post-transcriptional regulation, and competing endogenous RNA (ceRNA) activity through microRNA sponging, thereby influencing cell proliferation, differentiation, apoptosis, immune signaling, and metabolic adaptation [16,17,18]. Natural antisense lncRNAs are transcribed from the opposite strand of protein-coding genes, whereas lncRNAs are encoded by viral genomes and modulate host-cell gene expression [16]. CircRNAs and lncRNAs have emerged as important regulators of tumor initiation, progression, metastasis, and treatment resistance across a wide range of malignancies. In T-cell and NK-cell neoplasms, specific ncRNAs have been implicated in pathways involving TGF-β signaling, STAT3 activation, and glucose metabolism, and have demonstrated promise as diagnostic and prognostic biomarkers as well as potential therapeutic targets. Several have demonstrated promise as diagnostic and prognostic biomarkers, including minimally invasive circulating biomarkers, and may represent novel therapeutic targets [19,20,21,22,23,24].

This review focuses primarily on lncRNAs and circRNAs that have been experimentally implicated in the pathogenesis, diagnosis, prognosis, or therapeutic targeting of peripheral T-cell lymphoma (PTCL) and related T-cell and NK-cell neoplasms. Although numerous additional classes of non-coding RNAs participate in normal T-cell development, immune regulation, and other aspects of cellular homeostasis, their direct contribution to PTCL pathogenesis remains incompletely understood and requires further investigation. Recent advances have expanded the catalog of lymphoma-associated non-coding RNAs and highlighted their roles in immune regulation, treatment resistance, and precision oncology [25]. Here, we summarize the current understanding of the biological and clinical roles of lncRNAs and circRNAs in PTCL and related T-cell and NK-cell neoplasms, discuss their underlying molecular mechanisms, and highlight their growing significance as diagnostic and prognostic biomarkers, as well as their potential as therapeutic targets.

**Table 1:** Comparison of the World Health Organization Classification of Haematolymphoid Tumours, Fifth Edition (WHO-HAEM5), and the 2022 International Consensus Classification (ICC) of Mature T-Cell and Natural Killer (NK)-Cell Neoplasms.

Major Category	WHO-HAEM5 (2022) [6]	ICC (2022) [26]
**Mature T- and NK-cell leukemias**	T-PLL; T-LGLL; CLPD-NK; aggressive NK-cell leukemia	Included within mature T- and NK-cell neoplasms
**Primary cutaneous T-cell lymphomas**	Separate category	Included within mature T-cell neoplasms
**Intestinal T-cell and NK-cell lymphoid proliferations and lymphomas**	Separate category	Included within mature T-cell neoplasms
**Nodal TFH cell lymphomas**	Angioimmunoblastic type; follicular type; NOS	Recognized using similar terminology
**Anaplastic large cell lymphomas**	ALK-positive; ALK-negative; breast implant-associated	Recognized
**EBV-positive NK/T-cell lymphomas**	Extranodal NK/T-cell lymphoma, nasal type	Recognized
**Other peripheral T-cell lymphomas**	PTCL, NOS and related entities	Recognized

The fifth edition of the WHO-HAEM5 and the 2022 ICC provide complementary frameworks for the diagnosis of mature T-cell and natural killer (NK)-cell neoplasms. Although both systems recognize largely overlapping disease entities, they differ in terminology and organizational structure for selected categories, with notable distinctions involving nodal T-follicular helper (TFH) cell lymphomas, EBV-associated T- and NK-cell lymphoid proliferations, and cutaneous and intestinal T-cell lymphoma entities. The table summarizes the principal diagnostic categories and representative entities relevant to this review [5,6,26].

Abbreviations: AITL, angioimmunoblastic T-cell lymphoma; ALCL, anaplastic large cell lymphoma; ALK, anaplastic lymphoma kinase; CLPD-NK, chronic lymphoproliferative disorder of NK cells; EBV, Epstein–Barr virus; NOS, not otherwise specified; PTCL-NOS, peripheral T-cell lymphoma; T-LGLL, T-cell large granular lymphocytic leukemia; T-PLL, T-cell prolymphocytic leukemia.

Although protein-coding mRNAs constitute only a minor fraction of the human transcriptome, most transcribed RNA species are non-coding RNAs (ncRNAs). Structurally, ncRNAs can be broadly categorized into two groups: linear RNAs and circRNAs. CircRNAs are generated by back-splicing and include exonic circRNAs, exon–intron circRNAs, and circular intronic RNAs. They regulate gene expression through interactions with RNA-binding proteins, microRNA sponging, competing endogenous RNA activity, and, in some cases, translation of short peptides. Linear ncRNAs are subdivided into short ncRNAs and long non-coding RNAs (lncRNAs). Short ncRNAs include microRNAs (miRNAs), small nucleolar RNAs (snoRNAs), PIWI-interacting RNAs (piRNAs), and endogenous small interfering RNAs (siRNAs). Based on their genomic origin and orientation relative to neighboring genes, lncRNAs are commonly classified into sense, antisense, bidirectional, intronic, intergenic, and enhancer-associated transcripts [10,11,14].

## 2. Role of LncRNAs in the Pathogenesis of T-Cell Lymphoma

Aberrant lncRNA expression contributes to the initiation and progression of T-cell and NK-cell lymphomas by disrupting pathways regulating cellular proliferation, apoptosis, and differentiation [16] (Table 2). Although research in PTCL remains limited compared with B-cell malignancies, increasing evidence has identified lncRNAs involved in the pathogenesis of PTCL, extranodal NK/T-cell lymphoma, and anaplastic lymphoma kinase-negative (ALK−) anaplastic large cell lymphoma (ALCL) [16,17,20,27].

LncRNAs regulate gene expression through diverse mechanisms, including chromatin remodeling, transcriptional regulation, and interactions with chromatin-modifying complexes. One well-characterized example is metastasis-associated lung adenocarcinoma transcript 1 (MALAT1), which binds the Polycomb Repressive Complex 2 (PRC2) components enhancer of zeste homolog 2 (EZH2) and suppressor of zeste 12 (SUZ12), promoting H3K27 trimethylation and transcriptional repression of genes involved in cellular differentiation and growth control. These epigenetic changes contribute to aberrant T-cell proliferation and differentiation [28,29].

**Figure 2:**
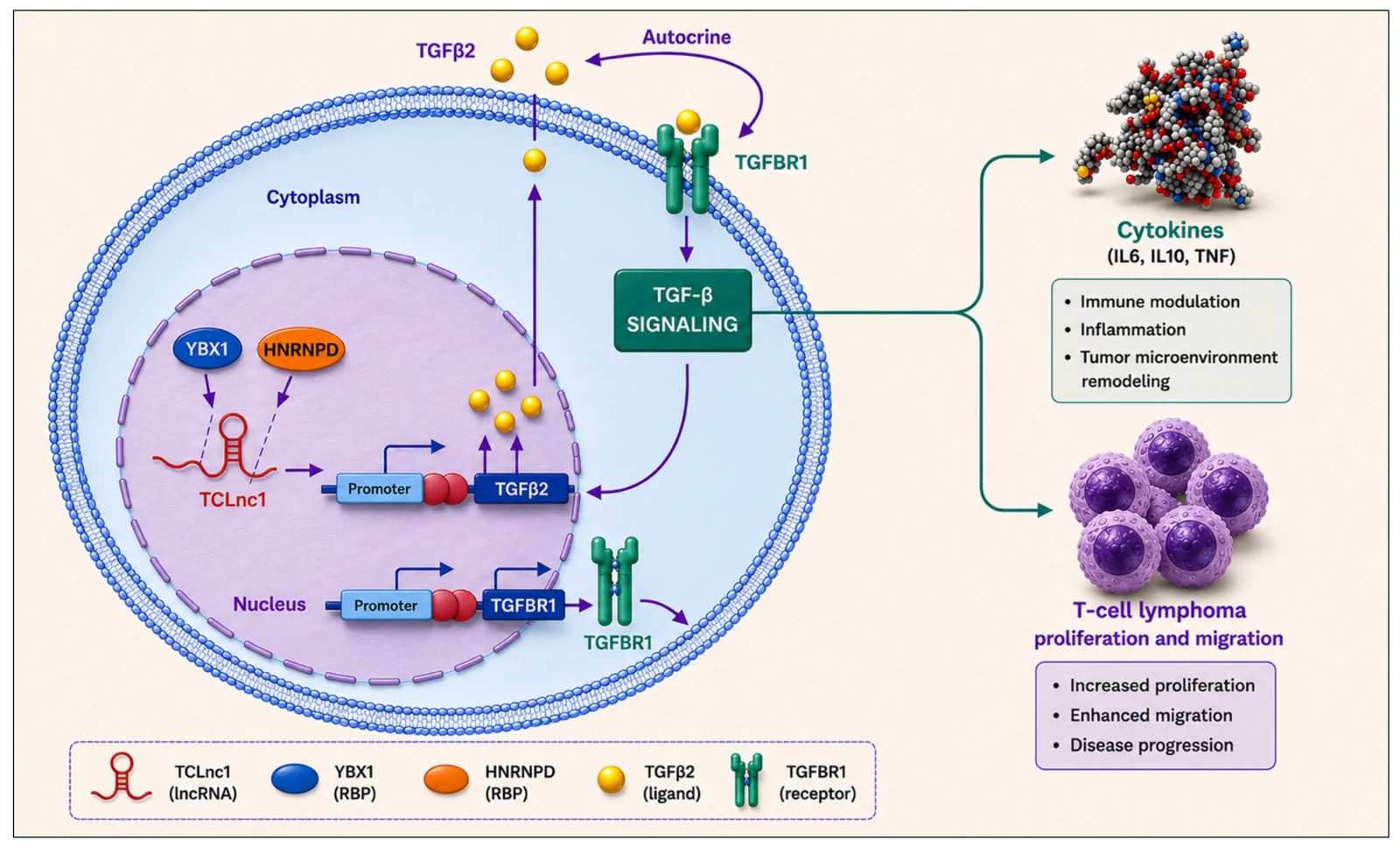
Mechanistic role of TCLlnc1 in peripheral T-cell lymphoma (PTCL).

Genome-wide transcriptomic profiling identified T-cell lymphoma-associated lncRNA 1 (TCLlnc1) as a novel oncogenic lncRNA that is highly expressed in PTCL [20]. Mechanistically, TCLlnc1 functions as a molecular scaffold for heterogeneous nuclear ribonucleoprotein D (HNRNPD) and Y-box binding protein 1 (YBX1), promoting expression of TGFB2 and TGFBR1 and subsequent activation of TGF-β signaling. These events enhance cytokine production, lymphoma-cell proliferation, and migration, thereby contributing to PTCL progression [20] (Figure 2).

**Table 2:** lncRNAs and circRNAs in T-Cell and NK-Cell Neoplasms.

RNA	Type	Lymphoma Subtype	Mechanism	Clinical Significance
**MALAT1**	lncRNA	T- and NK-cell lymphomas	Interacts with PRC2 (EZH2/SUZ12)	Poor prognosis [28]
**TCLlnc1**	lncRNA	PTCL	Scaffolds HNRNPD/YBX1; activates TGF-β signaling	Serum biomarker [20]
**MTAAT**	lncRNA	ALK-negative ALCL	Represses mitophagy and BNIP3	Therapeutic target [17]
**HOTAIR**	lncRNA	CTCL	Epigenetic regulation	Potential biomarker [30]
**ANRIL**	lncRNA	CTCL	Promotes proliferation and survival	Potential therapeutic target [30]
**MEG3**	lncRNA	CTCL	Tumor suppressor; induces apoptosis	Tumor suppressive role [30]
**circKIF4A**	circRNA	NK/T-cell lymphoma	Sponges miR-1231; upregulates PDK1/BCL11A	Poor OS and PFS [22]
**circADARB1**	circRNA	NK/T-cell lymphoma	Sponges miR-214-3p; activates STAT3	Plasma biomarker [21]
**circ-LAMP1**	circRNA	T-LBL	Regulates miR-615-5p/DDR2 axis	Diagnostic and therapeutic target [19]

Summary of biologically and clinically relevant long non-coding RNAs (lncRNAs) and circRNAs implicated in peripheral T-cell lymphomas (PTCLs) and related T-cell and natural killer (NK)-cell neoplasms. The table highlights representative ncRNAs, associated lymphoma subtypes, principal molecular mechanisms, and their potential diagnostic, prognostic, and therapeutic significance.

Abbreviations: PTCL, peripheral T-cell lymphoma; ALCL, anaplastic large cell lymphoma; CTCL, cutaneous T-cell lymphoma; T-LBL, T-cell lymphoblastic lymphoma; OS, overall survival; PFS, progression-free survival.

TCLlnc1 is an oncogenic long non-coding RNA overexpressed in PTCL. TCLlnc1 promotes PTCL progression through interaction with HNRNPD and YBX1. This ribonucleoprotein complex promotes transcription of TGFB2 and TGFBR1, resulting in activation of the transforming growth factor-β (TGF-β) signaling pathway. Downstream effects include increased cytokine production, enhanced cellular proliferation, migration, and progression of PTCL. Elevated serum TCLlnc1 levels are associated with advanced disease and inferior clinical outcomes [20].

## 3. Clinical Utility of lncRNAs as Biomarkers and Therapeutic Targets

Long non-coding RNAs (lncRNAs) are increasingly recognized as important contributors to the development and progression of T-cell and NK-cell lymphoid malignancies. Their diverse regulatory functions have generated considerable interest in their potential use as biomarkers and therapeutic targets. Among these molecules, MALAT1 has been associated with adverse clinical outcomes, with elevated expression levels correlating with shorter survival in patients with T-cell and NK-cell lymphomas [23]. Its oncogenic activity through PRC2-mediated epigenetic regulation provides a biological basis for its association with aggressive disease. Consequently, MALAT1 has potential clinical value as a prognostic biomarker and represents a promising therapeutic target in T- and NK-cell lymphomas [23,26] (Table 2).

TCLlnc1 is an oncogenic lncRNA identified as one of the most highly upregulated transcripts in peripheral T-cell lymphoma (PTCL). In a cohort of 70 PTCL patients treated with cyclophosphamide, doxorubicin, vincristine and prednisone (CHOP)-based chemotherapy, Zhao et al. demonstrated that TCLlnc1 expression was significantly increased in tumor tissues and that serum levels closely correlated with intratumoral expression. Elevated TCLlnc1 was associated with advanced stage, extranodal involvement, high International Prognostic Index (IPI), and inferior progression-free and overall survival. These clinicopathologic findings are consistent with the established oncogenic role of TCLlnc1 in promoting PTCL progression. For example, TCLlnc1 was markedly overexpressed across major PTCL subtypes and correlated with elevated serum levels of interleukin-6 (IL-6), IL-10, and tumor necrosis factor (TNF) [20]. These findings indicate that TCLlnc1 has potential clinical utility as a minimally invasive prognostic biomarker and as a therapeutic target in PTCL [24,25] (Figure 2).

Mularoni et al. identified a panel of lncRNAs capable of discriminating between ALK-positive and ALK-negative anaplastic large cell lymphoma (ALCL) subtypes [17]. Among these transcripts, MTAAT emerged as a key regulator associated with the ALK-negative phenotype. Functional studies demonstrated that MTAAT influences mitochondrial homeostasis through transcriptional regulatory mechanisms and modulation of mitophagy-related pathways [17]. Increased MTAAT expression was associated with enhanced cellular proliferation and disease aggressiveness, whereas loss of MTAAT promoted mitochondrial turnover and reduced tumor cell growth [17]. These findings suggest that MTAAT may serve both as a diagnostic biomarker and as a potential therapeutic target in ALK-negative ALCL. Furthermore, growing evidence indicates that mitochondrial fitness plays an important role in anti-tumor immune responses and may influence the efficacy of immunotherapeutic approaches, including chimeric antigen receptor-expressing T cell (CAR-T) therapy [31,32]. Collectively, these findings identify MTAAT as a promising diagnostic biomarker and potential therapeutic target in ALK-negative ALCL, particularly in the context of emerging immunotherapeutic strategies.

BCL2/adenovirus E1B 19 KDa protein 3 (BNIP3), a protein involved in apoptosis, autophagy and mitochondrial homeostasis, was identified as one of the downstream targets of MTAAT with increased upregulation upon interacting with MTAAT via chromatin rearrangement [17]. In certain tumors, the epigenetic silencing of BNIP3 is linked to increased cancer cell proliferation, unfavorable prognostic characteristics, and resistance to chemotherapy [33,34]. Supporting this, Mularoni et al. found a notable decrease in BNIP3 expression in a group of ALK^–^ ALCL patients, indicating that BNIP3 may have a tumor-suppressive role in ALCL [17]. These findings further support the BNIP3 pathway as a potential therapeutic target in ALK-negative ALCL.

Several lncRNAs have also demonstrated clinical relevance in cutaneous T-cell lymphoma (CTCL), where they function either as oncogenes or tumor suppressors depending on their expression pattern and molecular context. HOTAIR, PANDAR, ANRIL, and MALAT1 are frequently overexpressed and promote malignant behavior through epigenetic regulation, inhibition of apoptosis, and enhanced cellular proliferation. HOTAIR recruits chromatin-modifying complexes to alter gene expression, whereas ANRIL promotes cell-cycle progression and survival through repression of tumor suppressor pathways [30]. MALAT1 has been associated with adverse prognosis and may contribute to immune dysregulation and treatment resistance. In contrast, tumor-suppressive lncRNAs such as MEG3, LincRNA-p21, and CASC15-S are often downregulated [35]; restoration of their expression induces apoptosis and inhibits proliferation in CTCL cell lines [30] (Table 2). Collectively, these findings demonstrate the clinical significance of lncRNAs in the diagnosis, prognosis, and management of CTCL and highlight their potential as diagnostic and prognostic biomarkers, as well as therapeutic targets [28,30].

## 4. Role of CircRNA in the Pathogenesis of T-Cell Lymphoma

Circular RNAs (circRNAs) are a class of endogenous non-coding RNAs characterized by a covalently closed loop structure generated through back-splicing events. Unlike linear transcripts, circRNAs lack 5′ caps and 3′ poly(A) tails, which contributes to their enhanced stability. Increasing evidence suggests that circRNAs participate in diverse biological processes and contribute to cancer initiation, progression, metastasis, and treatment resistance. Their regulatory functions include modulation of gene expression, interaction with RNA-binding proteins, regulation of transcriptional networks, and sequestration of microRNAs through ceRNA mechanisms [22,36,37].

**Figure 3:**
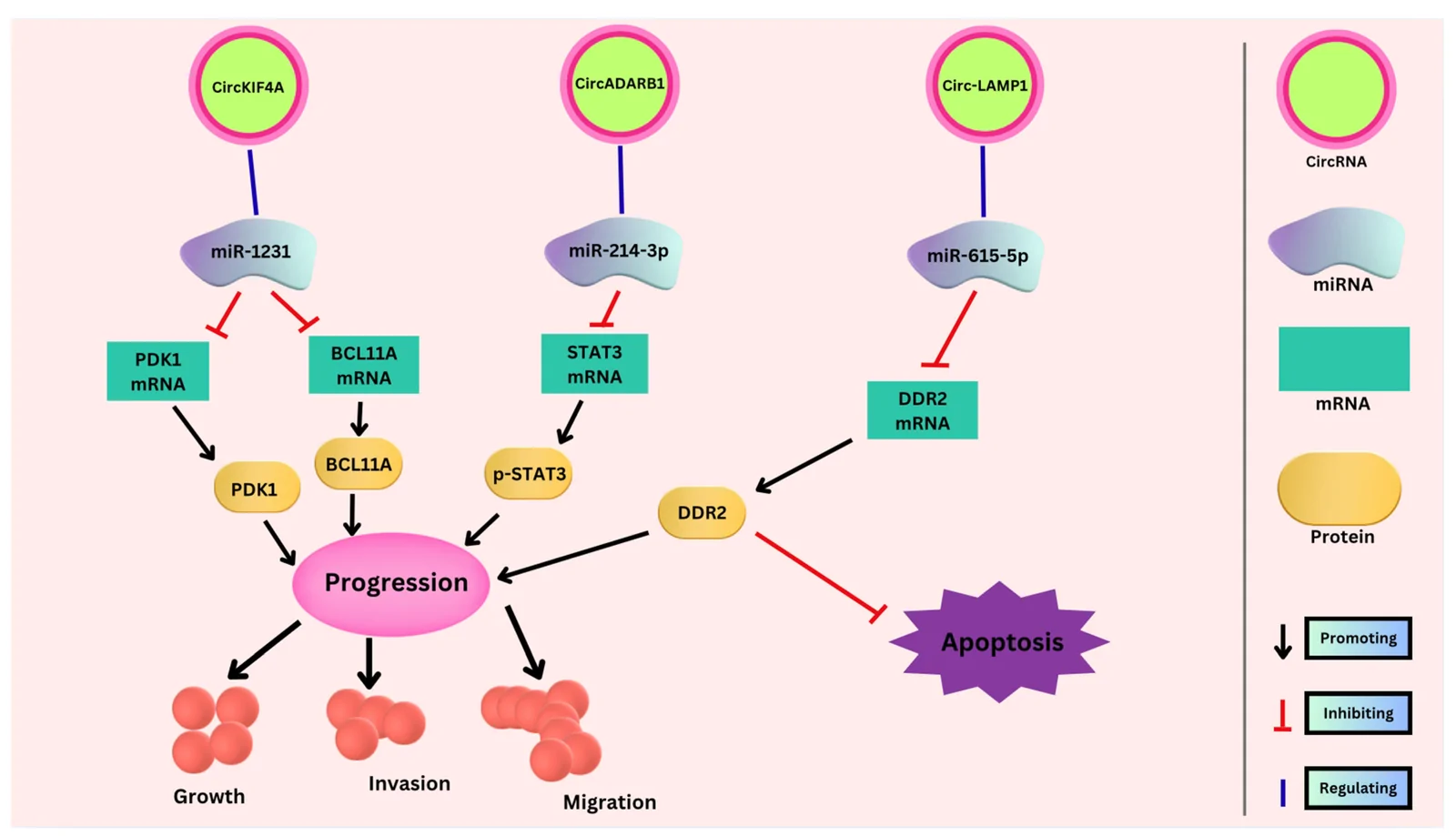
CircRNA-mediated oncogenic pathways in the pathogenesis of NKTL and T-LBL.

He et al. investigated the biological role of circKIF4A in NK/T-cell lymphoma (NKTL) and demonstrated that suppression of circKIF4A reduced glycolytic activity, as evidenced by decreased glucose utilization and lactate generation [22]. Mechanistically, circKIF4A was shown to function as a competing endogenous RNA capable of interacting with miR-1231. Through this interaction, circKIF4A may influence downstream targets including PDK1 and BCL11A, two genes implicated in malignant progression. Restoration of miR-1231 activity resulted in reduced expression of these targets, supporting the existence of a circKIF4A–miR-1231–PDK1/BCL11A regulatory axis in NKTL [22]. Collectively, these findings suggest that circKIF4A contributes to disease progression through modulation of cellular metabolism and oncogenic signaling pathways [22] (Figure 3).

Constitutive activation of the STAT3 pathway is a well-recognized driver of NK/T-cell lymphoma pathogenesis and has been implicated in tumor growth, survival, and disease progression [38,39,40,41]. Among the circRNAs associated with this signaling network, circADARB1 has attracted attention as a potential regulatory molecule. Mei et al. reported that circADARB1 is frequently overexpressed in NKTL and may promote tumor cell growth through modulation of the miR-214-3p/STAT3 signaling axis [21] (Figure 3). Furthermore, suppression of circADARB1 expression has been associated with reduced proliferative capacity and increased apoptotic activity in NKTL cells, highlighting its potential as a therapeutic target. Mutations in STAT3 can result in abnormally high expression of the activated form p-STAT3 with subsequent activation of the JAK/STAT3 pathway [42,43].

Another circRNA implicated in T-cell lymphoid malignancies is circ-LAMP1, which promotes T-cell lymphoblastic lymphoma (T-LBL) progression through a competing endogenous RNA mechanism involving miR-615-5p and its downstream target, discoidin domain receptor tyrosine kinase 2 (DDR2), a member of the receptor tyrosine kinase (RTK) family [19,44]. By sequestering miR-615-5p, circ-LAMP1 relieves miRNA-mediated repression of DDR2, resulting in increased DDR2 expression. Consistent with this mechanism, increased circ-LAMP1 expression was associated with enhanced cellular proliferation and reduced apoptotic activity, whereas disruption of the circ-LAMP1/miR-615-5p/DDR2 regulatory axis significantly impaired tumor cell growth [19] (Figure 3).

In NKTL, circKIF4A functions as a competing endogenous RNA (ceRNA) by sequestering miR-1231, thereby relieving miR-1231-mediated repression of PDK1 and BCL11A, which promotes glycolysis, tumor growth, invasion, and migration [22,44]. CircADARB1 is upregulated in NKTL and has been implicated in regulation of the miR-214-3p/STAT3 signaling axis. Current evidence suggests that circADARB1 sequesters miR-214-3p, resulting in increased STAT3 signaling and enhanced tumor growth; however, the precise molecular intermediates linking miR-214-3p to STAT3 activation remain incompletely defined [21,44]. In T-LBL, circ-LAMP1 functions as a ceRNA by sponging miR-615-5p, thereby relieving miR-615-5p-mediated repression of DDR2, which promotes cellular proliferation and suppresses apoptosis [44,45].

## 5. Clinical Utility of circRNAs as Biomarkers and Therapeutic Targets

Circular RNAs (circRNAs) have emerged as important regulators of tumor biology and may serve as diagnostic, prognostic, and therapeutic biomarkers in T-cell and NK-cell lymphomas. Through microRNA sponging, interactions with RNA-binding proteins, and modulation of oncogenic signaling pathways, circRNAs can influence cellular proliferation, apoptosis, metabolism, and treatment response [19,21,22].

In extranodal natural killer/T-cell lymphoma (NKTCL), several circRNAs have demonstrated significant clinical and biological relevance. He et al. showed that circKIF4A is markedly upregulated in NKTCL and is independently associated with inferior overall survival and progression-free survival [22]. He et al. showed that circKIF4A is markedly upregulated in NKTCL and is independently associated with inferior overall survival and progression-free survival [22]. Its established oncogenic role in promoting glycolysis and tumor growth provides a biological basis for its prognostic significance, supporting circKIF4A as a promising biomarker and potential therapeutic target [22].

Similarly, Mei et al. identified circADARB1 as significantly elevated in the plasma of patients with NKTCL, with higher levels associated with stable or progressive disease following treatment [21]. These findings support circADARB1 as a promising circulating biomarker for disease monitoring and a potential therapeutic target [21].

Circ-LAMP1 has also been found to be consistently upregulated in T-LBL tissues and cell lines [19]. Overexpression of circ-LAMP1 has been associated with disease progression, supporting its potential utility as both a biomarker and therapeutic target [19,44].

Collectively, these findings demonstrate that circRNAs contribute to the pathogenesis of T-cell and NK-cell lymphoid malignancies while also showing considerable promise as minimally invasive biomarkers for disease detection, prognostication, treatment monitoring, and the development of targeted therapeutic strategies. Although current evidence remains limited, ongoing research is likely to further define their clinical utility.

## 6. Conclusions

T-cell and NK-cell lymphoid malignancies comprise a diverse group of biologically heterogeneous diseases characterized by variable clinical behavior and often unfavorable outcomes. Despite advances in molecular classification and targeted therapeutic approaches, accurate diagnosis, risk stratification, and treatment selection face significant clinical challenges [16].

Recent advances in molecular profiling have substantially improved our understanding of disease pathogenesis and have identified novel diagnostic and therapeutic opportunities. Among these discoveries, long non-coding RNAs and circular RNAs have emerged as important regulators of gene expression and cellular signaling pathways involved in lymphoma initiation, progression, and treatment response [10,46]. Recent comprehensive reviews have further emphasized the expanding role of long non-coding RNAs as diagnostic biomarkers, prognostic indicators, and therapeutic targets in lymphoma [47].

The suppressive or oncogenic effects of specific circRNAs have primarily been assessed in vitro [44], highlighting the need for additional in vivo validation to further elucidate their pathological mechanisms. Advances in antisense oligonucleotides, small interfering RNAs, and other RNA-targeting therapeutics have accelerated the clinical translation of ncRNA-directed treatment strategies [48,49,50]. Novel delivery platforms, including nanoparticles and engineered extracellular vesicles, may further improve the specificity and clinical feasibility of ncRNA-targeted therapies [48].

Since completion of the literature search for this review, additional studies have further expanded the repertoire of clinically relevant non-coding RNAs in T-cell lymphoma. Recently, Wang et al. identified lncRNA IRENA as another oncogenic lncRNA involved in peripheral T-cell lymphoma progression by scaffolding ARHGEF1 and FMNL1 to activate RHOA GTPase/MAPK signaling, further highlighting the role of scaffold lncRNAs in T-cell lymphoma pathogenesis [51]. In parallel, Babin et al. demonstrated that circZBTB46 promotes crizotinib resistance in ALK-positive anaplastic large cell lymphoma through a miR-25-3p/PIP5K1C regulatory axis, highlighting its potential as a therapeutic target [52]. Collectively, these findings further emphasize the rapid pace of discovery and the growing translational potential of both lncRNAs and circRNAs in T-cell malignancies.

Future investigations should focus on validating ncRNA-based biomarkers in larger clinical cohorts, further elucidating the molecular mechanisms underlying their biological functions, and defining their utility in precision oncology approaches. Additional work is also required to establish the safety, efficacy, and clinical feasibility of ncRNA-directed therapeutic strategies and to determine how these approaches may complement existing diagnostic and treatment paradigms.

## Data Availability

No new data were created or analyzed in this study. Data sharing is not applicable to this article.
